# Establishment and preliminary application of object recognition system based on DeepLabCut

**DOI:** 10.3389/fnbeh.2026.1819151

**Published:** 2026-04-21

**Authors:** Cenfei Zhou, Yihua Sheng, Jing Xu, Xiaorui Peng, Zhujun Jia, Jianfei Wang, Yuanyun Zheng, Sidi Li

**Affiliations:** College of Pharmaceutical Sciences, Jishou University, Jishou, Hunan, China

**Keywords:** aging, artificial intelligence, cognition, DeepLabCut, object recognition analysis system, periodontitis

## Abstract

This study aimed to develop a DeepLabCut (DLC)-based object recognition analysis system for assessing rodent cognitive function and validate its application in natural aging and elderly periodontitis mouse models. The system’s hardware was constructed with a custom arena and high-definition industrial camera, and the DLC deep learning algorithm was trained to track five mouse body landmarks, enabling automatic quantification of 36 indicators across three categories: sniffing frequency, exploration duration, and novelty preference. The system subdivided exploratory behaviors by calibrating nose tip and body center, and set dynamic distance thresholds (1 cm, 1.5 cm, 2 cm) for the nose tip to capture fine-grained exploration. In the novel object recognition (NOR) and object location recognition (OLR) paradigms, traditional visual inspection failed to detect significant cognitive differences between young and aged mice, while the DLC system identified marked reductions in aged mice in the frequency and duration of body center and combined nose tip-body center exploration of the new object (2 cm away from the object), as well as corresponding novelty preference indices. In the elderly periodontitis models, traditional metrics showed increased nose tip exploration of the old object (2 cm away from the object) and reduced novelty preference in model mice; the DLC system further detected significantly elevated nose tip exploration frequency toward the old object (1.5 cm away from the object), accompanied by decreased frequency preference for exploration (1 cm away from the object). Collectively, this DLC-based system achieves sensitive, precise, and multidimensional quantification of mouse exploratory behavior, effectively distinguishing cognitive characteristics of aged and disease model mice. By overcoming the limitations of traditional methods, it captures subtle cognitive changes in aging and periodontitis models, screens key indicators for cognitive decline, and provides comprehensive behavioral evidence for elucidating the neural mechanisms underlying aging- and inflammation-associated cognitive impairment.

## 1 Introduction

Object recognition experiments are a widely used method in cognitive neuroscience and behavioral research, designed to assess cognitive abilities in animals (typically rodents) and humans, particularly non-spatial memory, recognition memory, and exploratory behavior (Seyedhosseini Tamijani et al., 2023; Blaser and Heyser, 2015; Antunes and Biala, 2012). This experiment is based on an individual’s preference for novel stimuli, meaning organisms tend to spend more time exploring novel objects than familiar ones (Antunes and Biala, 2012). Therefore, object recognition experiments infer an individual’s recognition memory capacity by measuring its exploration time toward novel objects. Currently, primary behavioral analysis equipment both domestically and internationally relies on principles such as computer vision capture, sensors, and video tracking for detection. However, these methods each possess certain limitations: computer vision capture primarily depends on changes in image pixels to extract animal contours, combined with digital image processing techniques, and cannot precisely capture specific animal behaviors (Fernandes et al., 2020); sensors are susceptible to interference from liquids (e.g., animal urine) and metals, potentially causing unstable signal transmission, while implanted tags may induce short-term stress responses in animals (Peleh et al., 2019). Video tracking primarily relies on background contrast to identify nose tip positions; object occlusions can lead to marker point loss and misjudgment, thereby compromising recognition accuracy (Benice and Raber, 2008; Spink et al., 2001). With the advancement of artificial intelligence technology, the DeepLabCut (DLC) algorithm has gained prominence and is widely applied in behavioral assessment. This algorithm utilizes AI to achieve automatic localization of markers in video footage (Ibañez et al., 2023; Lauer et al., 2022).

Cognitive decline in older adults poses a significant health challenge, and periodontitis is closely associated with cognitive impairment: the elderly are prone to periodontitis, and the chronic inflammation it induces may accelerate cognitive decline (Brahmbhatt et al., 2024; Ide et al., 2016). The interaction between these two conditions has become a research focus (Ding et al., 2018; Wu et al., 2026). In traditional object recognition experiments within this field, comprehensive and precise automated quantification of mouse behavior remains lacking. Using DLC v2.1.5, we developed a customized cognitive behavioral analysis system for object recognition in mice. Based on standard DLC feature point tracking technology, the system establishes an innovative analysis workflow and a customized behavioral analysis framework. Its features include multi-feature point collaborative decision-making, hierarchical distance thresholds, and head-direction filtering, which reduce false exploration events and enable precise quantification of cognitive behavior. This system was then applied to perform automated comprehensive analysis of behavioral performance in object recognition for aging and periodontitis models, which enables the analysis of cognition-related fine behaviors (such as exploration duration and sniffing frequency) and the automation of traditional metrics. By assessing changes in these fine behaviors, the system determines whether cognitive impairments have emerged in the disease models, mitigates the impact of accidental contact, and preliminarily explores cognitive strategies. This provides a methodological foundation for future object recognition experiments aimed at drug development for cognitive enhancement and research into the pathological mechanisms of cognitive impairment.

## 2 Materials and methods

### 2.1 Experimental animals

Twenty-eight male SPF-grade C57BL/6J mice were used, comprising 7 six-month-old, 14 fifteen-month-old mice,and 7 twenty-month-old animals. All mice were purchased from Hunan Slaike Jingda Laboratory Animal Co., Ltd. (License No. SCXK(Xiang)2019–0004). During the experiment, mice had free access to water and food, and strict adherence to relevant regulations for laboratory animal management was maintained. This study was reviewed and approved by the Ethics Committee of Jishou University (No. JSDX-2024-0101) and complied with the requirements of the Guidelines for the Management and Use of Laboratory Animals.

### 2.2 Reagents

*Porphyromonas gingivalis* (SHBCC D10892 = ATCC 33277) and *Fusobacterium nucleatum* (SHBCC D10462 = ATCC 25586) were purchased from Shanghai Bio-Sample Preservation Center; 4% carboxymethyl cellulose (Specification: 500 mL, Batch No.: P3137023) purchased from Shanghai Titan Technology Co., Ltd.

### 2.3 Hardware

Computer (Core i5 9400F, 2.9 GHz, NVIDIA GeForce RTX 2060 SUPER, 8 GB RAM, 500 GB HDD), object recognition experiment arena (dimensions: 50 cm × 50 cm × 50 cm, gray), and industrial camera (LouKe LRCP, 30 fps, resolution: 1920 × 1,080).

### 2.4 Quantification of metrics

Five body landmarks—the mouse’s nose tip, both ears, body center, and tail base—were manually annotated. Following annotation, DeepLabCut (DLC, v 2.1.5) was iteratively trained using an artificial neural network to construct an object recognition model capable of automatically tracking annotated landmark positions in images. Then, using the programming language Python as a metric quantification tool, the CSV file containing the marked point coordinates exported from DLC was automatically processed to calculate traditional metrics and new, refined object recognition metrics.

### 2.5 Model establishment

#### 2.5.1 Natural aging models

Fourteen male C57BL/6J mice were randomly selected, comprising seven 6-month-old (6 M) mice and seven 20-month-old (20 M) mice, representing young and aged mice, respectively.

#### 2.5.2 Elderly periodontitis models

Fourteen 15-month-old male C57BL/6J mice were randomly divided into a control group (Control) and a periodontitis model group (CAP), with 7 mice in each group. After 7 days of antibiotic treatment, drinking water was replaced with pure water for 3 days. A 20 μL suspension containing 1 × 10^9^ CFU of *Porphyromonas gingivalis* and *Fusobacterium nucleatum* in 2% carboxymethylcellulose was administered to mice via gastric lavage. The control group received sterile 2% carboxymethylcellulose solution. Following colonization by oral pathogens, the cementoenamel junction-alveolar bone crest (CEJ-ABC) distance significantly increased in the periodontitis model group compared to the control group, indicating alveolar bone resorption and successful establishment of the periodontitis models (Jiang et al., 2025). Object recognition behavioral experiments 112 were conducted.

### 2.6 Object recognition experiment

#### 2.6.1 Novel object recognition paradigm (NOR)

This experiment comprises three phases: adaptation, familiarization, and testing. During adaptation, mice are exposed to the apparatus and allowed to explore for 10 min. Following a 30-min interval, the familiarization phase commences. During familiarization, mice freely explore two identical objects in different corners of the arena for 10 min. After a 30-min interval, the 10-min test phase begins. One original object is placed in its familiar position, while the other is replaced with a novel object. The apparatus is thoroughly cleaned with 70% ethanol after each trial to minimize odor effects on mouse behavior.

#### 2.6.2 Object location recognition paradigm (OLR)

The location recognition experiment was conducted the day after the novel object recognition test. The familiarization phase of the location recognition experiment was identical to that of the novel object recognition test. During the test phase, one of the two objects was moved to a new location (novel), while the other remained unchanged (familiar). Each mouse was exposed to the objects for 10 min. After each trial, the experimental apparatus was repeatedly cleaned with 70% ethanol to minimize the influence of odors on mouse behavior.

### 2.7 Statistical methods

Statistical analysis was performed using SPSS 27.0 software. Quantitative data were expressed as mean ± standard deviation (x ± s). Prior to conducting between-group comparisons, the Shapiro–Wilk normality test and Levene’s test for homogeneity of variances were performed. For data that were normally distributed and had homogeneous variances, an independent samples *t*-test was used; for data that were normally distributed but had heterogeneous variances, a Welch-corrected t-test was used; and for data that did not meet the normality assumption, the Mann–Whitney U test was used. To control for Type I errors resulting from multiple comparisons, *p*-values for all intergroup comparisons were corrected using the Benjamini–Hochberg (BH) error rate correction in Python 3.9, and the corrected *q*-values were reported; *q < 0.05 was considered statistically significant. Effect sizes were also calculated, with Cohen’s d used for the independent samples t-test and the ranked correlation coefficient r for the Mann–Whitney U test. Furthermore, a paired *t*-test was used to compare the consistency between manual observations and the results of this system; Pearson correlation analysis was employed to evaluate the degree of association between the two methods, with the correlation coefficient *r* and *p*-value reported; a *p*-value < 0.05 was considered statistically significant.

## 3 Results

### 3.1 Hardware of the deep learning-based object recognition analysis system

**Figure 1:**
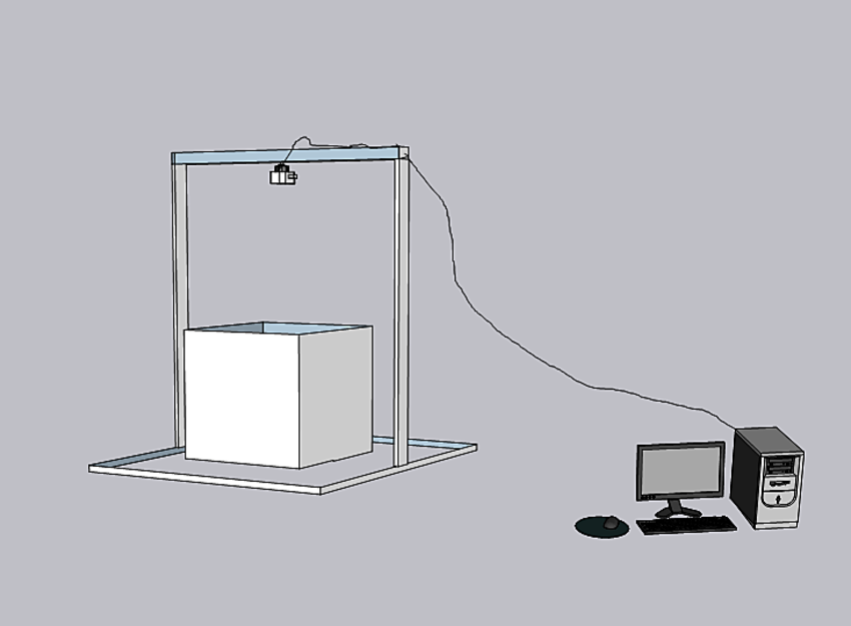
Hardware of the object recognition analysis system.

The system hardware comprises an object recognition competition box, a high-definition industrial camera, and a computer. The industrial camera is positioned directly above the device to capture overhead images of mice. The computer records and stores images, trains and establishes the deep learning network model, tracks marker points, and automates metric quantification (Figure 1).

### 3.2 Artificial neural network

**Figure 2:**
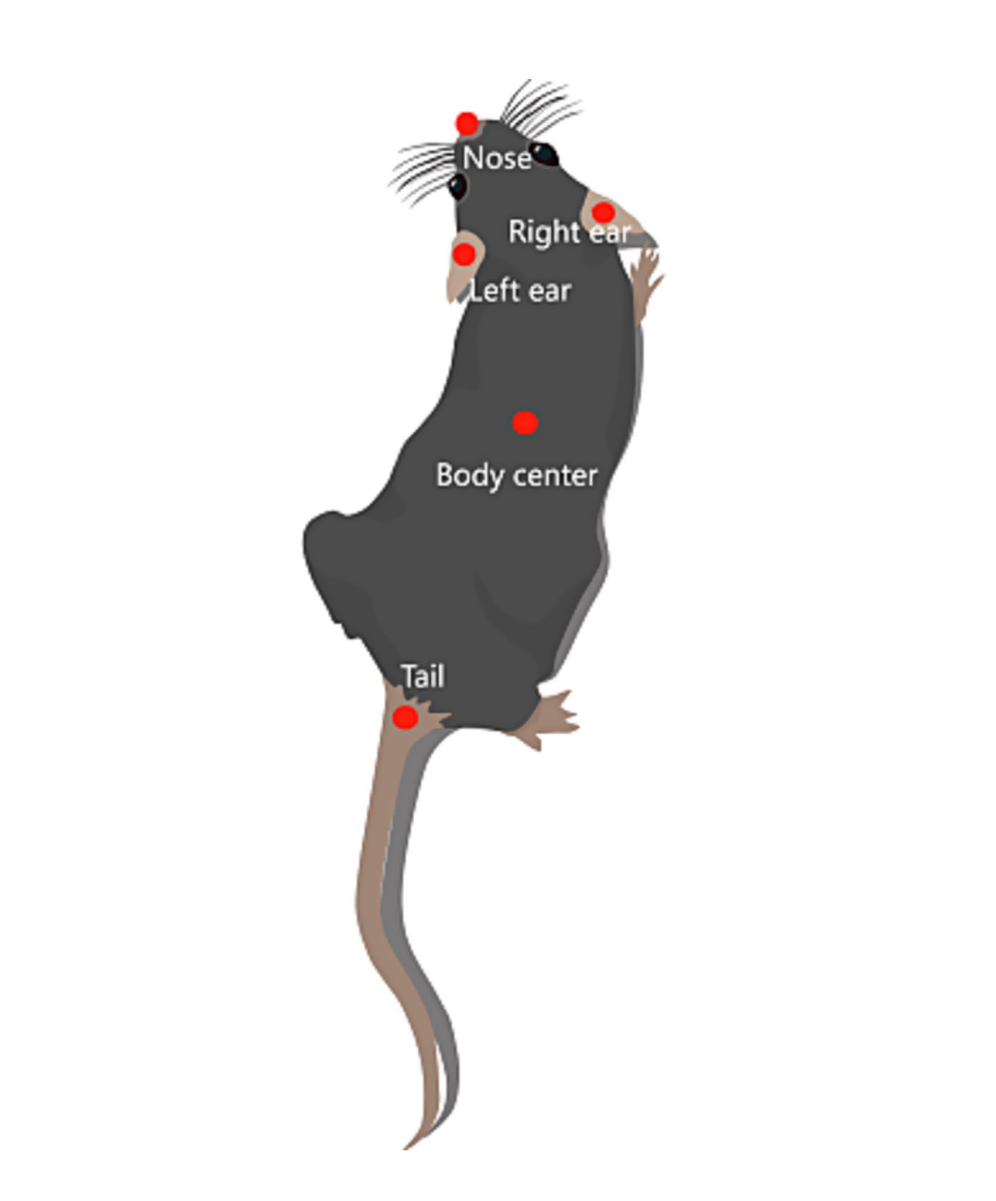
Markers used for DLC artificial neural network training.

Mice images for object recognition were manually annotated with 5 body region markers: nose tip, left ear, right ear, body center, and tail base (Figure 2). After annotation, iterative training of the artificial neural network algorithm was conducted in DLC.

### 3.3 Object recognition behavioral evaluation metrics

This system first achieves automatic quantification of traditional metrics for object recognition methods, including the frequency and duration of nose tip explorations of new and old objects (2 cm away from the object), as well as the preference for the frequency and duration of nose tip explorations of objects (2 cm away from the object). The specific definitions of the traditional behavioral indicators are detailed in Supplementary Table S1.

Based on DeepLabCut (DLC) landmark tracking, this system characterizes the physiological basis of behavioral perception (integrating both visual detection and tactile contact) in rodents using novel object recognition (NOR) behavior recorded by a single overhead camera. According to standardized behavioral definitions in the literature, valid object exploration is strictly defined as active nose-directed approach toward an object with close-range attention or physical contact, excluding mere passing or locomotion around objects. We further established a graded dynamic nose-to-object distance threshold system comprising 1 cm, 1.5 cm, and 2 cm thresholds. These three thresholds have been applied in previous literature and form a complementary gradient capable of reliably distinguishing between specific cognitive processing and non-specific motor activity (Aggleton et al., 2010; Liu et al., 2025; Antunes and Biala, 2012). Based on this, the system can automatically quantify a comprehensive set of novel behavioral metrics: frequency and duration of nose tip explorations of new and old objects (within 1 cm and 1.5 cm from the object), preference for frequency and duration of nose tip explorations of objects (within 1 cm and 1.5 cm from the object), frequency and duration of body center explorations of new and old objects (2 cm away from the object), preference for frequency and duration of body center explorations of objects (2 cm away from the object), frequency and duration of nose tip and body center explorations of new and old objects (2 cm away from the object), preference for frequency and duration of nose tip and body center explorations of objects (2 cm away from the object), frequency and duration of nose tip touches to new and old objects, preference for frequency and duration of nose tip touches to objects. The specific definitions of the refined behavioral indicators are given in Supplementary Table S2.

### 3.4 System validation

Manual visual recording and analysis by the object recognition fine-behavior analysis system were conducted on videos from the object recognition experiment, focusing on the frequency and duration of nose tip explorations toward new and old objects (2 cm away from the object), as well as the preference for the frequency and duration of nose tip explorations toward objects (2 cm away from the object). Results indicate no significant difference between the two methods (all *p* > 0.05), demonstrating high similarity (the full comparison results between manual scoring and the automated NOR analysis system are presented in Supplementary Table S3). Furthermore, a significant correlation was observed between the results obtained from manual visual observation and those from the object recognition fine-behavior analysis system (Table 1). These findings demonstrate that the object recognition fine-behavior analysis system can accurately analyze object recognition experimental data.

**Table 1:** The results of correlation analysis between visual observation and NOR analysis system.

Indicator	Correlation coefficient	*p*
Frequency of exploring a new object by the nose tip (2 cm away from the object)	0.924	<0.005
Frequency of exploring an old object by the nose tip (2 cm away from the object)	0.965	<0.001
Duration of exploring a new object by the nose tip (2 cm away from the object) (s)	0.981	<0.001
Duration of exploring an old object by the nose tip (2 cm away from the object) (s)	0.967	<0.001
Frequency preference for exploring objects with the tip of the nose (2 cm away from the object)	0.890	<0.01
Duration preference for exploring objects with the tip of the nose (2 cm away from the object)	0.837	<0.05

The data conformed to a normal distribution; therefore, Pearson’s correlation coefficient was employed for analysis. Here, *p* indicates the significance of the correlation.

### 3.5 Behavioral analysis of object recognition in aging models

#### 3.5.1 Changes in NOR behavior in the aging models

**Figure 3:**
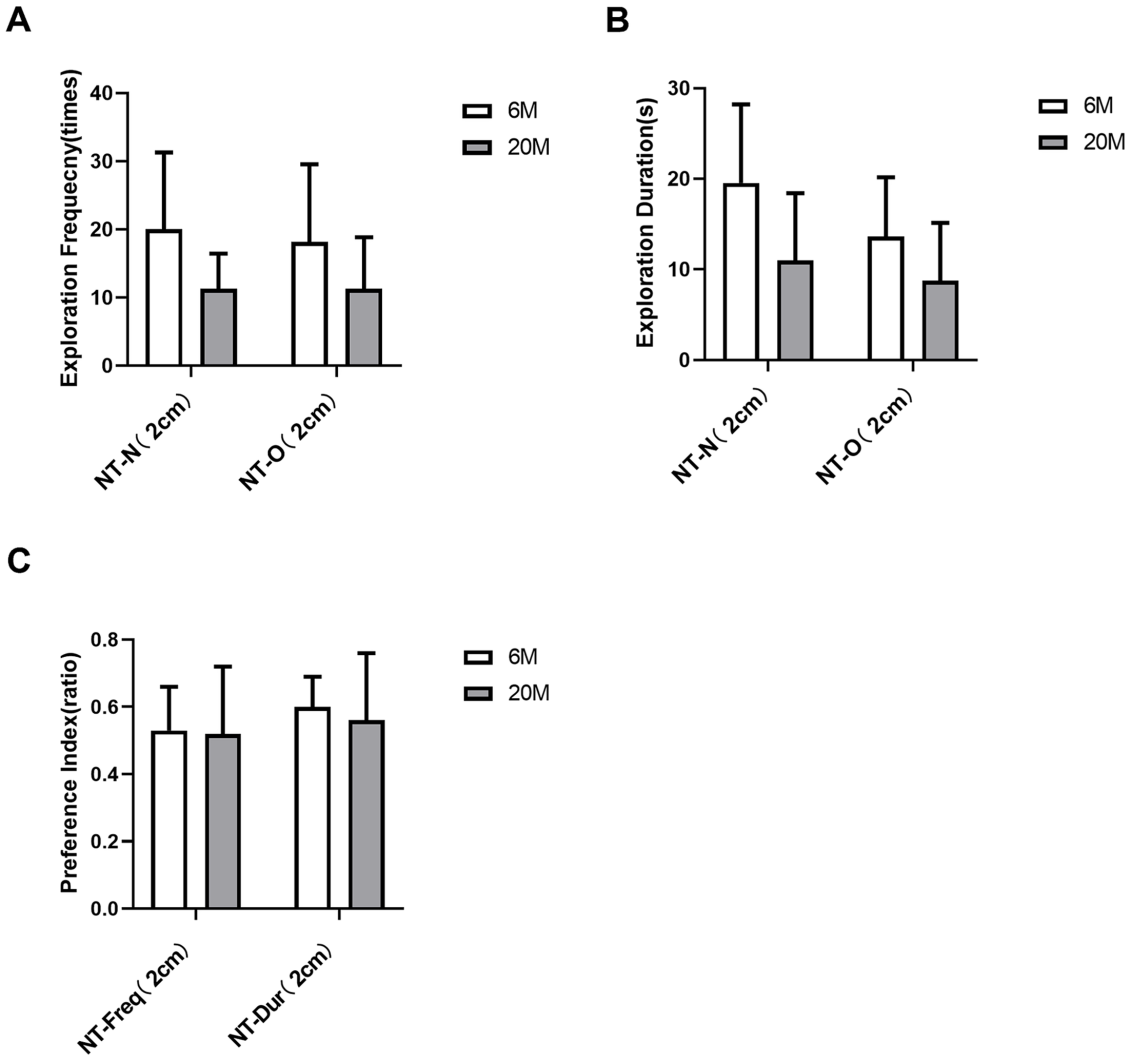
Analysis of traditional behavioral measures in the novel object recognition (NOR) task in natural aging models. **(A)** Exploration frequency for the new object [NT-N(2 cm)] and the old object [NT-O (2 cm)] with the nose tip at a 2 cm distance threshold; **(B)** Exploration duration for the new object [NT-N (2 cm)] and the old object [NT-O (2 cm)] with the nose tip at a 2 cm distance threshold; **(C)** Preference indices for nose tip exploration frequency [NT-Freq-Pref (2 cm)] and exploration duration [NT-Dur-Pref (2 cm)] toward the new object at the 2 cm distance threshold. White bars represent the 6-month-old young control group, and gray bars represent the 20-month-old aged group (*n* = 6 mice per group). Error bars indicate mean ± standard deviation (SD). Intergroup statistical comparisons were performed using the two-sided Student’s *t*-test for independent samples (for normally distributed data) or the Mann–Whitney U test (for non-normally distributed data). To control the Type I error rate and false discovery rate (FDR), all 6 behavioral measures in this figure were uniformly subjected to Benjamini–Hochberg (BH) stepwise correction; statistical significance was defined as a corrected *q*-value < 0.05 (*q < 0.05). No statistically significant differences were observed between groups for any of the measures (all corrected *q*-values > 0.05). NT, Nose Tip; N, New Object; O, Old Object; Freq, Exploration Frequency; Dur, Exploration Duration; Pref, Preference Index; numbers in parentheses indicate the distance threshold from the object (cm).

Compared to the 6 M group, mice in the 20 M group showed a trend toward reduced frequency and duration of exploring the new and old objects by the nose tip (2 cm away from the object) in traditional behavioral metrics (Figures 3A,B), though this was not statistically significant (all *q* > 0.05 after Benjamini-Hochberg correction). This indicates that differences in novel object recognition pattern between 6 M and 20 M mice could not be detected by visual observation (Figure 3C).

**Figure 4:**
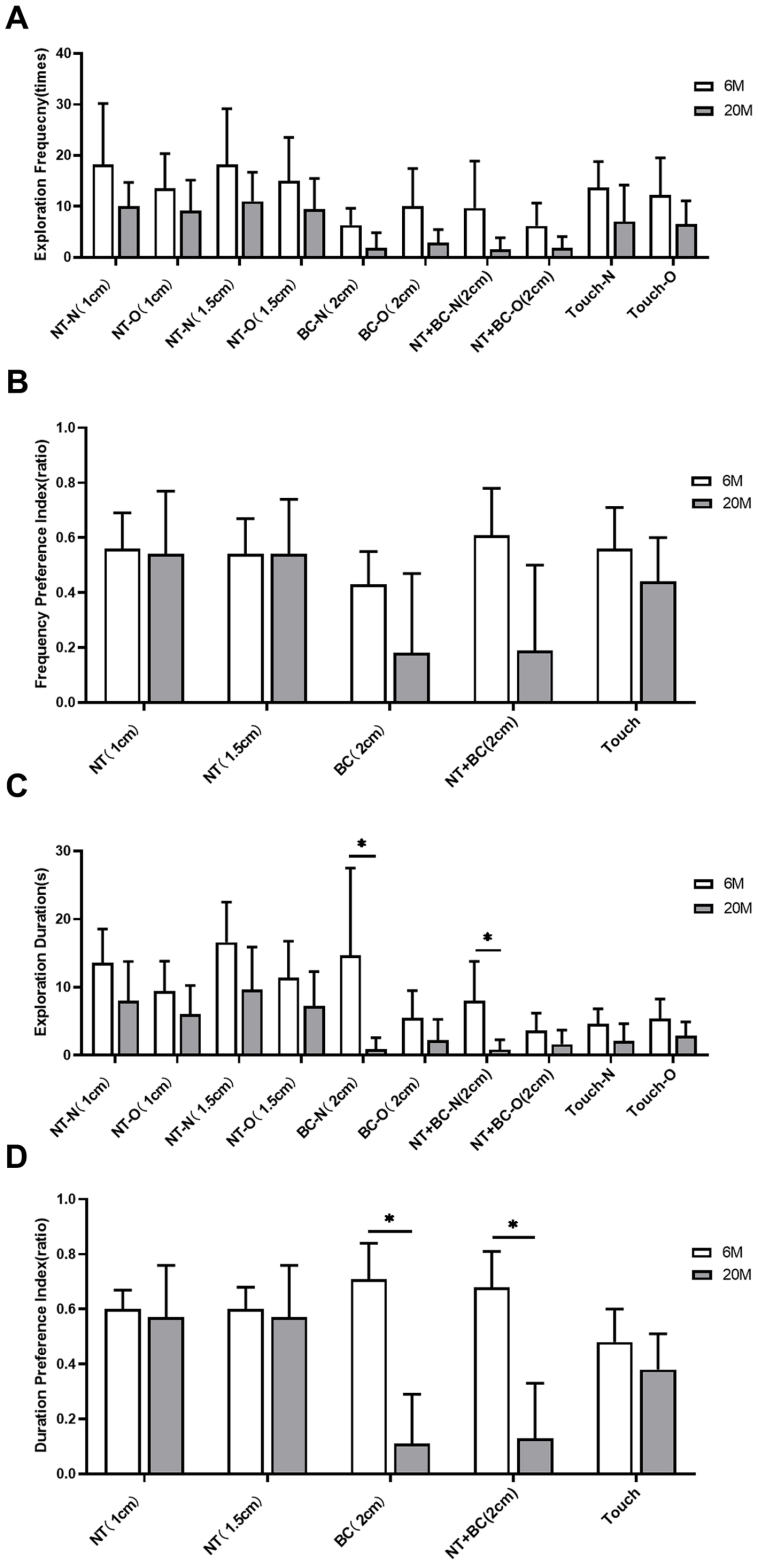
Analysis of fine-motor behavioral indicators in the novel object recognition (NOR) task in natural aging models. **(A)** Exploration frequency for the new object (N) and old object (O) measured by nose tip (NT), body center (BC), nose tip + body center (NT + BC), and touch (Touch) at different distance thresholds; **(B)** Preference indices of exploration frequency toward the new object (N) for NT, BC, NT + BC, and Touch at different distance thresholds; **(C)** Exploration duration for the new object (N) and old object (O) based on NT, BC, NT + BC, and Touch at different distance thresholds; **(D)** Preference indices of exploration duration toward the new object for NT, BC, NT + BC, and Touch at different distance thresholds. White bars represent the 6-month-old young control group, and gray bars represent the 20-month-old aged group (*n = 6 mice per group*). *Error bars in*dicate mean ± standard deviation (SD). Intergroup statistical comparisons were performed using the two-sided Student’s *t*-test for independent samples (for normally distributed data) or the Mann–Whitney U test (for non-normally distributed data). To control the Type I error rate and false discovery rate (FDR), all 30 behavioral measures in this figure were subjected to Benjamini–Hochberg (BH) stepwise correction; statistical significance was defined as a corrected *q*-value < 0.05 (*q < 0.05). After correction, only some indicators showed statistically significant differences between groups (see the Results section of the main text for specific *q*-values); the remaining indicators showed no statistically significant differences between groups (all corrected *q*-values were >0.05). NT, Nose Tip; BC, Body Center; N, New Object; O, Old Object; Touch, Nose Tip Contact with Object; numbers in parentheses indicate distance thresholds from the object (cm).

In this fine-motor behavior analysis system, for both frequency-based and response preference-based measures, the exploration frequency of mice in the 20 M group showed a downward trend compared to the 6 M group (Figure 4A),the preference for exploring the object with the body center (2 cm away from the object) and the preference for exploring the object with the nose tip and body center (2 cm away from the object) were significantly reduced (Figure 4B), but none of these differences were statistically significant (all *q* > 0.05 after Benjamini-Hochberg correction). Regarding duration-related measures, compared to the 6 M group, the 20 M group showed a significant reduction in the duration of body center exploration of the new object (2 cm away from the object) (*q* = 0.023, *r* = 0.231; Figure 4C)and the duration of nose tip and body center exploration of the new object (2 cm away from the object) (*q* = 0.023, *r* = 0.231; Figure 4C). Additionally, regarding duration preference measures, compared with the 6 M group, the 20 M group showed a marked reduction in the duration preference for exploring the object with the body center (2 cm away from the object) (*q* = 0.023, *r* = 0.245; Figure 4D) and the duration preference for exploring the object with the nose tip and body center (2 cm away from the object) (*q* = 0.023, *r* = 0.245; Figure 4D). These results suggest that mice in the aging models exhibit cognitive impairment-like behaviors.

#### 3.5.2 Changes in OLR behavior in aging models

**Figure 5:**
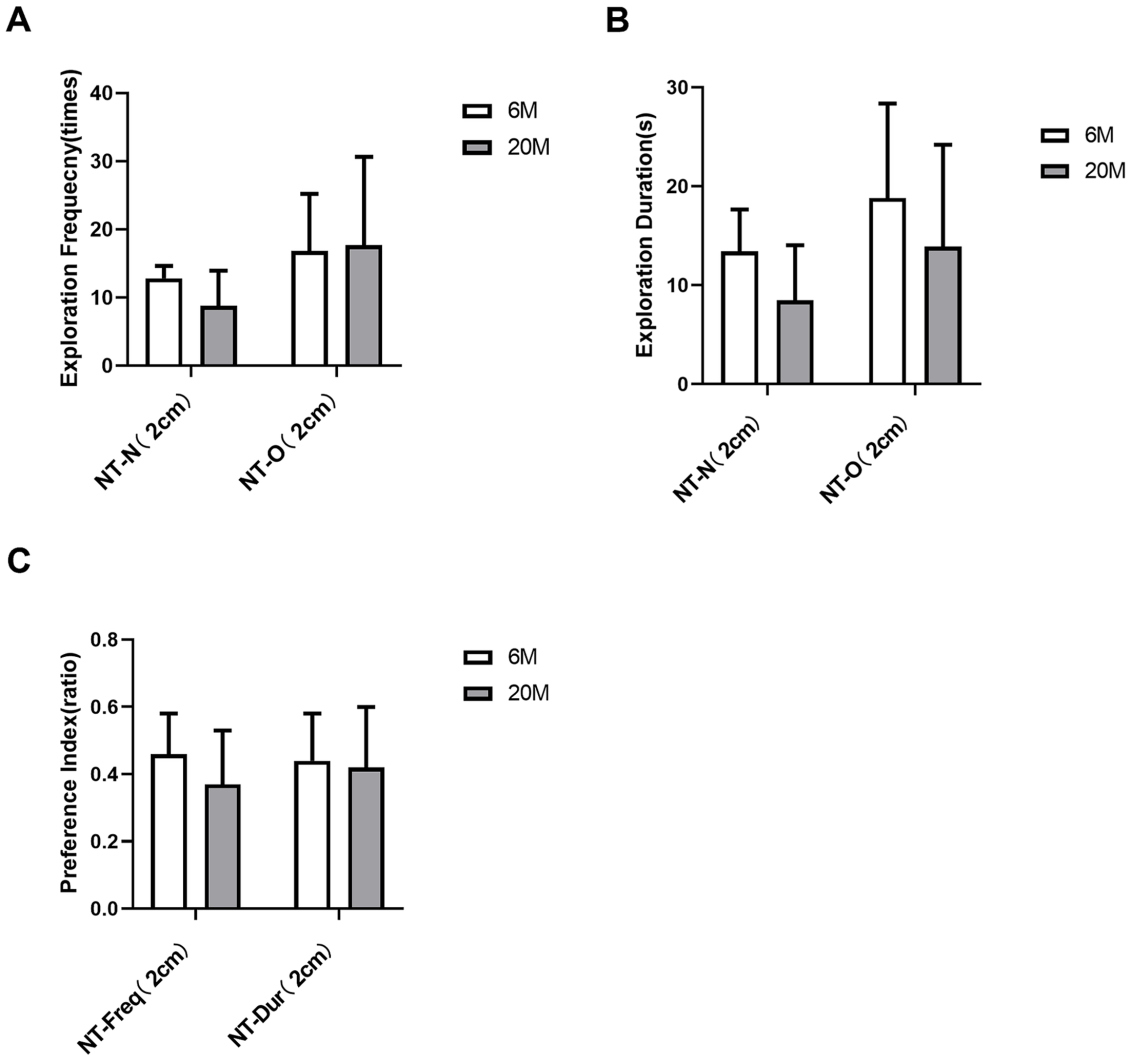
Analysis of traditional behavioral measures in the object location recognition (OLR) task in natural aging models. **(A)** Exploration frequency for objects in a new location [NT-N (2 cm)] and objects in an old location [NT-O (2 cm)] with the nose tip at a 2-cm distance threshold; **(B)** Exploration duration for the new-location object [NT-N (2 cm)] and the old-location object [NT-O (2 cm)] with the nose tip at a 2 cm distance threshold; **(C)** Preference indices for nose tip exploration frequency [NT-Freq-Pref (2 cm)] and exploration duration [NT-Dur-Pref (2 cm)] toward the object in the new location at the 2 cm distance threshold. White bars represent the 6-month-old young control group, and gray bars represent the 20-month-old aged group (*n* = 6 mice per group). Error bars indicate mean ± standard deviation (SD). Intergroup statistical comparisons were performed using the two-sided Student’s *t*-test for independent samples (for normally distributed data) or the Mann–Whitney U test (for non-normally distributed data). To control the Type I error rate and false discovery rate (FDR), all 6 behavioral measures in this figure were uniformly subjected to Benjamini–Hochberg (BH) stepwise correction; statistical significance was defined as a corrected q-value < 0.05 (*q < 0.05). No statistically significant differences were observed between groups for any of the measures (all corrected *q*-values > 0.05). NT, Nose Tip; N, New Location Object; O, Old Location Object; Freq, Exploration Frequency; Dur, Exploration Duration; Pref, Preference Index; numbers in parentheses indicate the distance threshold from the object (cm).

Compared to the 6 M group, mice in the 20 M group showed a trend toward reduced frequency and duration of exploring the new and old objects by the nose tip (2 cm away from the object) in traditional behavioral metrics (Figures 5A,B), though this was not statistically significant (all *q* > 0.05 after Benjamini-Hochberg correction). This suggests that cognitive differences between the two groups cannot be distinguished by visual observation (Figure 5C).

**Figure 6:**
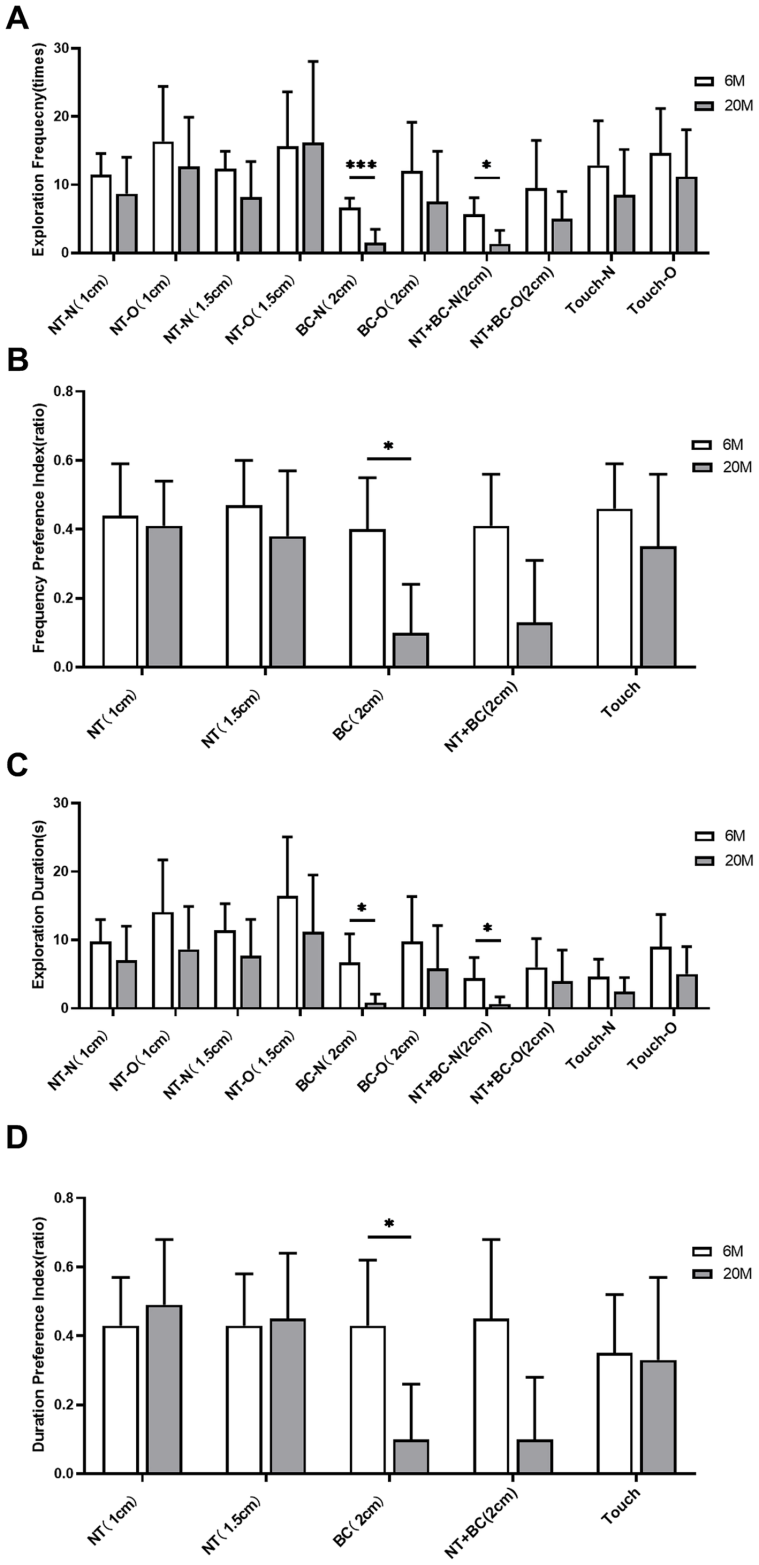
Analysis of fine-motor behavioral indicators in the object location recognition (OLR) task in natural aging models. **(A)** Exploration frequency for objects in new (N) and old (O) locations measured by nose tip (NT), body center (BC), nose tip + body center (NT + BC), and touch (Touch) at different distance thresholds; **(B)** Preference indices for exploration frequency of objects in a new location (N) and an old location (O) based on NT, BC, NT + BC, and touch at different distance thresholds; **(C)** Exploration duration for objects in the new location (N) and old location (O) based on NT, BC, NT + BC, and touch at different distance thresholds; **(D)** Preference indices for exploration duration of objects in new locations for NT, BC, NT + BC, and touch at different distance thresholds. White bars represent the 6-month-old young control group, and gray bars represent the 20-month-old aged group (*n* = 6 *mice per group*). *Error bars in*dicate mean ± standard deviation (SD). Intergroup statistical comparisons were performed using the two-sided Student’s *t*-test for independent samples (for normally distributed data) or the Mann–Whitney *U* test (for non-normally distributed data). To control the Type I error rate and false discovery rate (FDR), all 30 behavioral indicators in this figure were subjected to Benjamini–Hochberg (BH) stepwise correction; statistical significance was defined as a post-correction *q*-value < 0.05 (^*^q < 0.05, ^**^q < 0.01, ^***^q < 0.001). After correction, only differences in a subset of indicators between groups were statistically significant (see the results section in the main text for specific *q*-values); differences in the remaining indicators between groups were not statistically significant (all post-correction *q*-values > 0.05). NT, Nose Tip; BC, Body Center; N, New Location Object; O, Old Location Object; Touch, Nose Tip Contact with Object; numbers in parentheses indicate distance thresholds from the object (cm).

Among the frequency-based measures in this analysis system, compared with the 6 M group, the 20 M group showed a significant reduction in the exploration frequency of the new object by the body center (2 cm away from the object) (*q* = 0.000, Cohen’s d = 3.043; Figure 6A) and by the nose tip and body center (2 cm away from the object) (*q* = 0.035, Cohen’s d = 1.964; Figure 6A). Regarding frequency preference metrics, the 20 M group showed a significant decrease in the frequency preference for exploring objects with the body center (2 cm away from the object) (*q* = 0.035, *r* = 1.965; Figure 6B), suggesting impaired spatial recognition memory in the aged model mice. Meanwhile, regarding duration-related measures, compared with the 6 M group, the 20 M group showed a significant reduction in the duration of body center exploration of the new object (2 cm away from the object) (*q* = 0.040, *r* = 0.242; Figure 6C) and the duration of nose tip and body center exploration of the new object (2 cm away from the object) (*q* = 0.040, *r* = 0.228; Figure 6C). Regarding duration preference measures, the 20 M group showed a significant decrease in the duration preference for exploring the objects with the body center (2 cm away from the object) (*q* = 0.040, Cohen’s d = 1.904; Figure 6D). These results suggest that the spatial recognition memory function is impaired in aged model mice.

### 3.6 Analysis of memory-impairment-like behavior in periodontitis models

**Figure 7:**
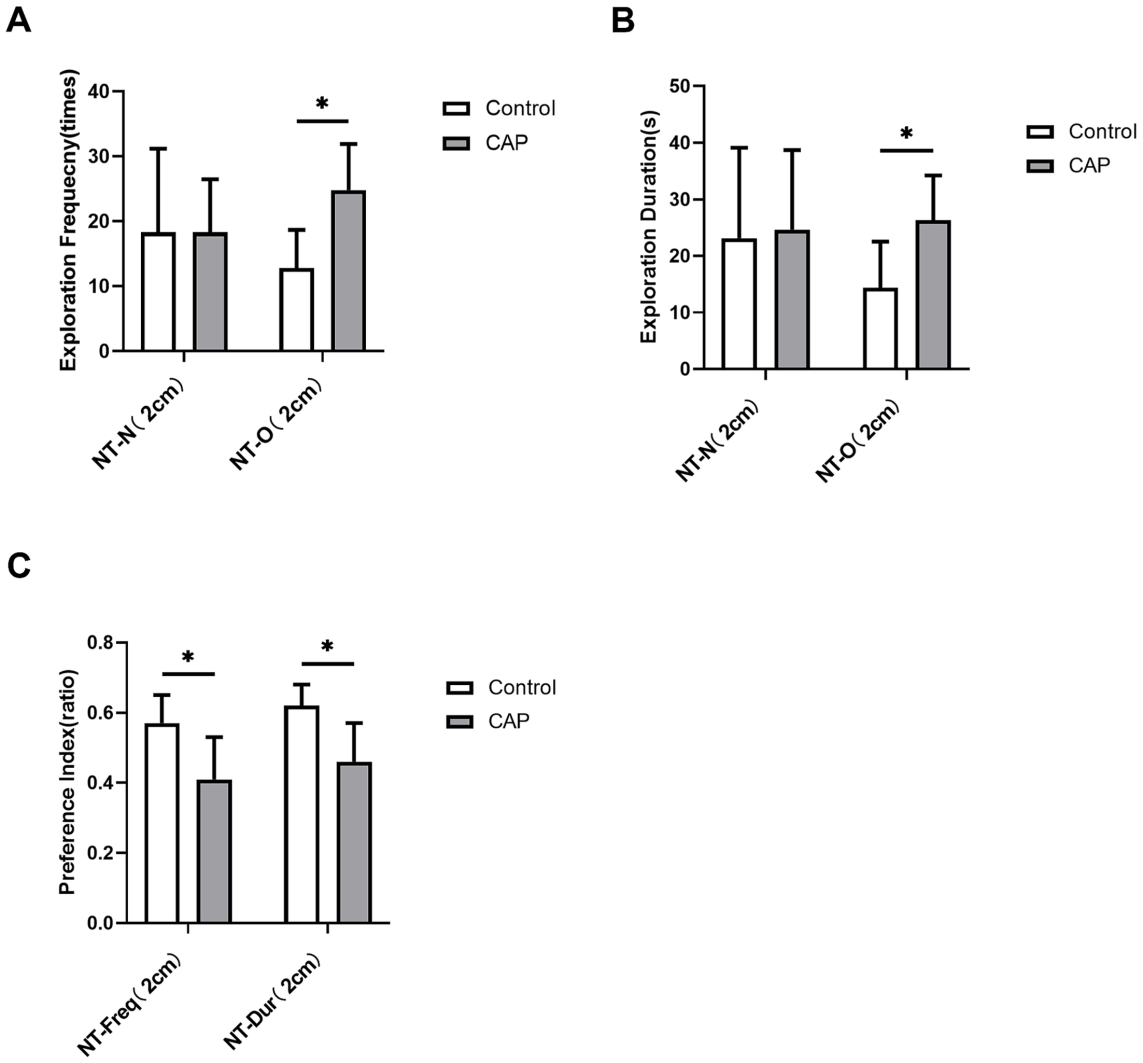
Analysis of traditional behavioral measures in the novel object recognition (NOR) task in periodontitis models. **(A)** Exploration frequency for the new object [NT-N (2 cm)] and the old object [NT-O (2 cm)] with the nose-tip at a 2 cm distance threshold; **(B)** Exploration duration for the new object [NT-N (2 cm)] and the old object [NT-O (2 cm)] with the nose-tip at a 2 cm distance threshold; **(C)** Preference indices for nose-tip exploration frequency [NT-Freq-Pref (2 cm)] and exploration duration [NT-Dur-Pref (2 cm)] toward the new object at the 2 cm distance threshold. White bars represent the control group (Control), and gray bars represent the periodontitis model group (CAP) (*n* = 6 mice per group). Error bars indicate mean ± standard deviation (SD). Intergroup statistical comparisons were performed using the two-sided Student’s *t*-test for independent samples (for normally distributed data) or the Mann–Whitney *U* test (for non-normally distributed data). To control the Type I error rate and false discovery rate (FDR), all 6 behavioral indicators in this figure were subjected to Benjamini–Hochberg (BH) stepwise correction; statistical significance was defined as a corrected *q*-value < 0.05 (^*^q < 0.05, as indicated in the figure). After correction, differences between groups were statistically significant for some indicators (see the results section in the main text for specific *q*-values). NT, Nose Tip; N, New Object; O, Old Object; Freq, Exploration Frequency;Dur, Exploration Duration; Pref, Preference Index; CAP, *Porphyromonas gingivalis*-induced periodontitis models; numbers in parentheses indicate the distance threshold from the object (cm).

Compared with the Control group, mice in the CAP group exhibited significantly increased frequency (*q* = 0.042, Cohen’s d = −1.848; Figure 7A) and duration (*q* = 0.042, Cohen’s d = −1.488; Figure 7B) of exploring the old object by the nose tip (2 cm away from the object) in traditional behavioral metrics. While both the frequency preference for exploring objects with nose tip (2 cm away from the object) (*q* = 0.042, Cohen’s d = 1.51; Figure 7C) and the duration preference for exploring objects with nose tip (2 cm away from the object) were significantly reduced (*q* = 0.042, Cohen’s d = 1.67; Figure 7C). No significant differences were observed in the other two conventional indicators. This suggests that mice in the CAP group exhibited a tendency toward increased exploratory activity toward the new object (Figure 7).

**Figure 8:**
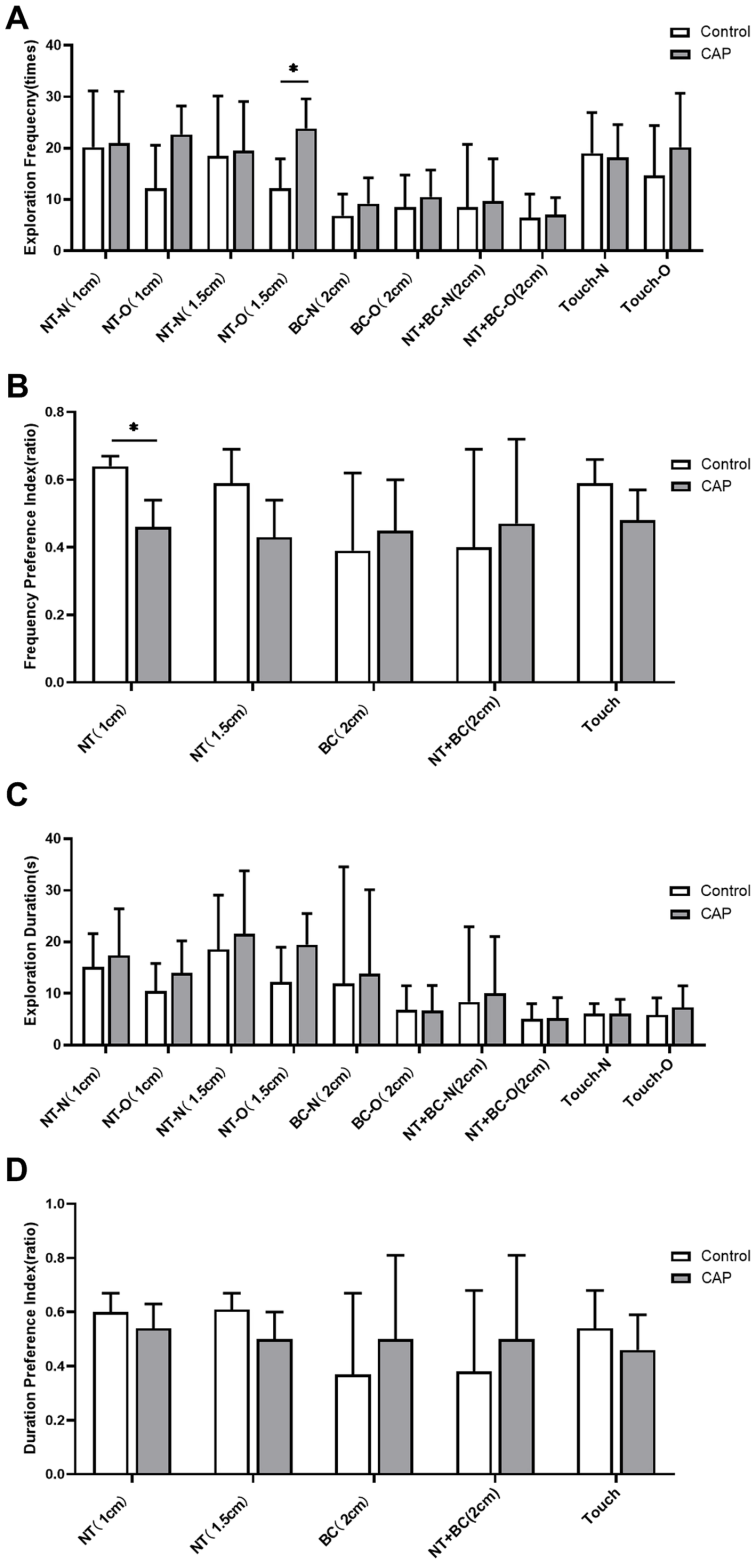
Analysis of fine-motor behavioral indicators in the novel object recognition (NOR) task in periodontitis models. **(A)** Exploration frequency for the new object (N) and old object (O) measured by nose tip (NT), body center (BC), nose tip + body center (NT + BC), and touch (Touch) at different distance thresholds; **(B)** Preference indices for exploration frequency toward the new object (N) based on NT, BC, NT + BC, and touch at different distance thresholds; **(C)** Exploration duration for the new object (N) and old object (O) based on NT, BC, NT + BC, and touch at different distance thresholds; **(D)** Preference indices of exploration duration toward the new object for NT, BC, NT + BC, and touch at different distance thresholds. White bars represent the control group, and gray bars represent the periodontitis group (*n* = 6 mice per group). Error bars indicate mean ± standard deviation (SD). Intergroup statistical comparisons were performed using the two-sided Student’s *t*-test for independent samples (for normally distributed data) or the Mann–Whitney *U* test (for non-normally distributed data). To control the Type I error rate and false discovery rate (FDR), all 30 behavioral measures in this figure were subjected to Benjamini–Hochberg (BH) stepwise correction; statistical significance was defined as a corrected *q*-value < 0.05 (^*^q < 0.05, as indicated in the figure). After correction, only differences in a subset of indicators between groups were statistically significant (see the results section in the main text for specific *q*-values); differences in the remaining indicators between groups were not statistically significant (all post-correction *q*-values > 0.05). NT, Nose Tip; BC, Body Center; N, New Object; O, Old Object; Touch, Nose Tip Contact with Object; numbers in parentheses indicate distance thresholds from the object (cm).

In the detailed behavioral analysis system, regarding frequency-based measures, CAP group mice showed a significant increase in the exploration frequency of the old object by the nose tip (1.5 cm away from the object) compared to the Control group (*q* = 0.045, Cohen’s d = −2.032; Figure 8A); regarding frequency preference measures, the preference indices for nose tip exploration of objects (1 cm away from the object) were significantly reduced in the CAP group (*q* = 0.030, Cohen’s d = 2.912; Figure 8B), whereas no statistically significant differences were observed in duration and duration preference measures (Figures 8C,D). These results indicate that periodontitis-induced cognitive impairment is more pronounced in the dimension of close-range fine exploration; model mice exhibited significantly impaired preference for actively identifying novel objects, while their tendency to explore familiar objects was abnormally enhanced.

## 4 Discussion

The author developed an automated object recognition analysis system based on the DLC without modifying the core tracking algorithm. By simultaneously tracking multiple body locations—including the nose tip, both ears, body center, and tail base, the system achieves traditional object recognition metrics while automatically quantifying 36 evaluation indicators across three dimensions: exploration duration, frequency, and preference. This enables high-precision, multidimensional quantification of exploratory behavior in mouse object recognition experiments, providing richer behavioral evidence for cognitive function assessment. Unlike traditional methods relying solely on the nose tip, this analysis system enables more precise definition of exploratory behavior by selecting different body locations (nose tip, body center, or both nose tip and body center) as calibration points for collaborative analysis. This effectively distinguishes exploratory from non-exploratory behavior, minimizing interference from accidental touches. Additionally, the system innovatively evaluates overall perception and fine cognitive abilities through distance parameters (1 cm, 1.5 cm, 2 cm) between the nose tip and objects, objectively quantifying subtle exploratory behaviors unattainable by conventional methods. We applied this system to aging and periodontitis models to validate the system’s effectiveness and sensitivity, and used detailed behavioral metrics to capture subtle cognitive changes in the model mice.

Traditional object recognition experiments rely on visual observation by the experimenter, and generally use the nose tip entering the 2-cm zone surrounding the object as the criterion for determining exploratory behavior. However, in this study, under both the NOR and OLR experimental conditions, the traditional metric of the nose tip exploring the objects (2 cm away from the object) showed no statistically significant difference in this aging models. Considering that nose tip exploration is susceptible to interference from nonspecific exploration (accidental contact behavior) (Ibañez et al., 2023), this study further introduced body center-based exploration as an indicator of overall cognitive state and combined nose tip and body center as a dual-point reference indicator. Results revealed that in both NOR and OLR paradigms, compared to the 6 M group, the 20 M group exhibited significantly reduced duration and duration preference of body-center-centered exploration of the new object (2 cm away from the object), nose-tip-body-center-combined exploration of the new object (2 cm away from the object). Additionally, the 20 M group exhibited significantly reduced the frequency of body-center object exploration (2 cm away from the object) and nose-tip-body-center-combined exploration of the new object (2 cm away from the object) in OLR compared to the 6 M group. This aligns with previous findings of age-related cognitive decline in learning and memory in mice (Hendrickx et al., 2022; Jiang et al., 2023; Dean et al., 1981) and better captures the progressive cognitive deterioration typical of aging (Jiang et al., 2023), suggesting that multidimensional behavioral metrics can reduce false negatives associated with the unidimensional nose-only approach. It is worth noting that while using body center markers alone as an indicator for exploratory behavior is more sensitive than traditional methods, it remains difficult to distinguish between active exploratory behavior and non-specific spatial approach. To address this, the system innovatively introduces a 2-centimeter exploration radius centered on the nose and body center, utilizing three key head landmarks (nose and both ears) to establish a head-triangle orientation determination mechanism: a head orientation vector is constructed based on these three landmarks. Only when the head is clearly oriented toward an object and meets the behavioral definition is the action recorded as valid exploratory behavior. This mechanism effectively eliminates interference from non-specific behaviors—such as animals merely passing near an object without actively recognizing it—significantly improving the specificity and accuracy of exploration behavior detection, thereby ensuring that the quantified results more accurately reflect the animal’s true cognitive state. Furthermore, aging is closely associated with periodontitis, and advancing age significantly increases the risk and severity of periodontitis (Ide et al., 2016). As a chronic peripheral inflammatory disorder, periodontitis continuously releases pro-inflammatory cytokines including IL-1β, IL-6, and TNF-*α*, accompanied by the entry of *Porphyromonas gingivalis* and its virulence factors into the circulation (Ding et al., 2018). These mediators and pathogenic factors can cross the blood–brain barrier and act preferentially on cognition-related brain regions such as the hippocampus, cerebral cortex, and motor cortex (Ide et al., 2016; Ding et al., 2018; Wu et al., 2026). They activate microglia and induce persistent neuroinflammation and oxidative stress (Ding et al., 2018), which in turn reduce VGluT1 levels, suppress glutamatergic transmission, impair synaptic plasticity, and promote abnormal aggregation of Ser129-phosphorylated α-synuclein (Ide et al., 2016). Collectively, these pathological changes damage neuronal function, disrupt learning, memory, and exploratory behavior, and ultimately exacerbate cognitive impairment (Ide et al., 2016; Ding et al., 2018; Wu et al., 2026). To further validate the applicability of this system in models of disease-related cognitive impairment, this system was applied to an aged (15-month-old) periodontitis model. Among traditional indicators, the frequency and duration of nose tip exploration of unfamiliar objects (2 cm away from the object) significantly increased; while the frequency and duration of nose tip exploration of objects (2 cm away from the object) showed a marked decline. This suggests impaired object recognition memory in aged periodontitis mice, with periodontitis may exacerbate age-related cognitive decline (Brahmbhatt et al., 2024; Ma et al., 2023), further demonstrating the accuracy of this system in automatically quantifying traditional indicators. Notably, this system detected significantly increased frequency of nose tip exploration of the old object (1.5 cm away from the object) in aged periodontitis mice compared to age-matched controls. The frequency preference for exploring objects with the tip of the nose (1 cm away from the object) was markedly reduced. This suggests that traditional metrics may only reflect overt features of cognitive impairment, whereas close-range exploratory behavior better reflects the mouse’s active recognition and memory retrieval processes. By reducing interference from non-specific exploratory behaviors, it more accurately captures subtle cognitive changes in mice,may serve as a sensitive behavioral indicator for detecting subtle cognitive deficits.

In summary, the customized object recognition and analysis system built on the DLC platform overcomes the technical limitations of traditional object recognition methods and identifies cognitive abnormalities that were previously undetectable by conventional approaches. This system utilizes standard DLC for automatic tracking of landmarks and innovatively employs Python to perform in- depth analysis of DLC point coordinates, independently developing and constructing 36 innovative behavioral indicators. Furthermore, through the system’s detailed behavioral indicator analysis alone, it can accurately identify cognitive impairment-like behaviors in the models, thereby enhancing the sensitivity of cognitive anomaly detection. Furthermore, the system incorporates three gradient distance thresholds (1 cm, 1.5 cm, and 2 cm) and includes a head orientation mechanism. This dual-innovation design effectively prevents accidental touches from interfering with exploratory behavior, thereby increasing the method’s accuracy and reliability and resolving detection biases caused by interfering factors in traditional methods. To further validate the system’s applicability, it was applied to models of natural aging and aged periodontitis. In the natural aging models, the system was able to detect cognitive abnormalities in aged mice that were previously undetectable by traditional object recognition methods; through the analysis of the system’s detailed behavioral indicators alone, cognitive impairment-like behaviors in aged mouse model could be accurately identified; in the aged periodontitis models, it was found that the nose-tip exploration metrics (1 cm and 1.5 cm away from the object) capture more nuanced animal exploration behaviors compared to the traditional nose-tip exploration metric (2 cm away from the object). These metrics can more sensitively detect subtle changes in cognitive impairment, potentially providing a reference for the behavioral assessment of cognitive dysfunction. In conclusion, this system can identify specific, sensitive behavioral indicators associated with cognitive impairment across different stages of various cognitive disorder models and conduct comprehensive, detailed assessments. This may provide a more refined behavioral assessment tool for evaluating the efficacy of cognitive-enhancing drugs and holds promise for future application in the screening of cognitive-enhancing drugs and research into the pathological mechanisms underlying cognitive impairment.

## Data Availability

The original contributions presented in the study are included in the article/Supplementary material, further inquiries can be directed to the corresponding author.

## References

[ref1] Aggleton J. P. Albasser M. M. Aggleton D. J. Poirier G. L. Pearce J. M. (2010). Lesions of the rat perirhinal cortex spare the acquisition of a complex configural visual discrimination yet impair object recognition. Behav. Neurosci. 124, 55–68. doi: 10.1037/a0018320, 20141280 PMC2834571

[ref2] Antunes M. Biala G. (2012). The novel object recognition memory: neurobiology, test procedure, and its modifications. Cogn. Process. 13, 93–110. doi: 10.1007/s10339-011-0430-z, 22160349 PMC3332351

[ref3] Benice T. S. Raber J. (2008). Object recognition analysis in mice using nose-point digital video tracking. J. Neurosci. Methods 168, 422–430. doi: 10.1016/j.jneumeth.2007.11.002, 18096240

[ref4] Blaser R. Heyser C. (2015). Spontaneous object recognition: a promising approach to the comparative study of memory. Front. Behav. Neurosci. 9:183. doi: 10.3389/fnbeh.2015.00183, 26217207 PMC4498097

[ref5] Brahmbhatt Y. Alqaderi H. Chinipardaz Z. (2024). Association between severe periodontitis and cognitive decline in older adults. Life (Basel) 14:1589. doi: 10.3390/life14121589, 39768299 PMC11678878

[ref6] Dean R. L. 3rd Scozzafava J. Goas J. A. Regan B. Beer B. Bartus R. T. (1981). Age-related differences in behavior across the life span of the C57BL/6J mouse. Exp. Aging Res. 7, 427–451. doi: 10.1080/03610738108259823, 7333338

[ref7] Ding Y. Ren J. Yu H. Yu W. Zhou Y. (2018). *Porphyromonas gingivalis*, a periodontitis causing bacterium, induces memory impairment and age-dependent neuroinflammation in mice. Immun. Ageing 15:6. doi: 10.1186/s12979-017-0110-7, 29422938 PMC5791180

[ref8] Fernandes A. F. A. Dórea J. R. R. Rosa G. J. M. (2020). Image analysis and computer vision applications in animal sciences: an overview. Front. Vet. Sci. 7:551269. doi: 10.3389/fvets.2020.551269, 33195522 PMC7609414

[ref9] Hendrickx J. O. De Moudt S. Calus E. De Deyn P. P. Van Dam D. De Meyer G. R. Y. (2022). Age-related cognitive decline in spatial learning and memory of C57BL/6J mice. Behav. Brain Res. 418:113649. doi: 10.1016/j.bbr.2021.113649, 34728276

[ref10] Ibañez V. Bohlen L. Manuella F. Mansuy I. Helmchen F. Wahl A.-S. (2023). EXPLORE: a novel deep learning-based analysis method for exploration behaviour in object recognition tests. Sci. Rep. 13:4249. doi: 10.1038/s41598-023-31094-w, 36918658 PMC10014875

[ref11] Ide M. Harris M. Stevens A. Sussams R. Hopkins V. Culliford D. . (2016). Periodontitis and cognitive decline in Alzheimer's disease. PLoS One 11:e0151081. doi: 10.1371/journal.pone.0151081, 26963387 PMC4786266

[ref12] Jiang W. R. Wu W. Yang L. J. Yang W. Tian Q. Yao Z. H. (2023). Alteration of cognitive function in aging and Alzheimer's disease mice is related to dysfunction of the Neuroimmune system. J. Alzheimer's Dis 94, 815–839. doi: 10.3233/JAD-230292, 37334607

[ref13] Jiang Y. Xu L. Zhao X. Shen H. Qiu C. He Z. . (2025). *Porphyromonas gingivalis*-induced periodontitis promotes neuroinflammation and neuronal loss associated with dysfunction of the brain barrier. Front. Cell. Infect. Microbiol. 15:1559182. doi: 10.3389/fcimb.2025.1559182, 40552124 PMC12183281

[ref14] Lauer J. Zhou M. Ye S. Menegas W. Schneider S. Nath T. . (2022). Multi-animal pose estimation, identification and tracking with DeepLabCut. Nat. Methods 19, 496–504. doi: 10.1038/s41592-022-01443-0, 35414125 PMC9007739

[ref15] Liu C. Zhang C. Chen L. Liu X. Wu J. Sun Y. . (2025). Lingo1 in the hippocampus contributes to cognitive dysfunction after anesthesia and surgery in aged mice. Int. J. Biol. Sci. 21, 595–613. doi: 10.7150/ijbs.98376, 39781463 PMC11705636

[ref16] Ma X. Shin Y. J. Yoo J. W. Park H. S. Kim D. H. (2023). Extracellular vesicles derived from *Porphyromonas gingivalis* induce trigeminal nerve-mediated cognitive impairment. J. Adv. Res. 54, 293–303. doi: 10.1016/j.jare.2023.02.006, 36796586 PMC10703712

[ref17] Peleh T. Bai X. Kas M. J. H. Hengerer B. (2019). RFID-supported video tracking for automated analysis of social behaviour in groups of mice. J. Neurosci. Methods 325:108323. doi: 10.1016/j.jneumeth.2019.108323, 31255597

[ref18] Seyedhosseini Tamijani S. M. Beirami E. Ghazvini H. Rafaiee R. Nazeri M. Razavinasab M. (2023). A review on the disruption of novel object recognition induced by methamphetamine. Addict. Health. 15, 289–297. doi: 10.34172/ahj.2023.1307, 38322487 PMC10843358

[ref19] Spink A. J. Tegelenbosch R. A. J. Buma M. O. S. Noldus L. P. J. J. (2001). The EthoVision video tracking system—a tool for behavioral phenotyping of transgenic mice. Physiol. Behav. 73, 731–744. doi: 10.1016/S0031-9384(01)00530-3, 11566207

[ref20] Wu Z. Zhang Y. Wang L. Yi Y. Dai B. Chen H. . (2026). Periodontitis and systemic diseases: insights into the correlation, mechanisms, and clinical implications. Front. Immunol. 17:1777955. doi: 10.3389/fimmu.2026.1777955, 41890752 PMC13013007

