# Supplementary material for: Establishment and preliminary application of object recognition system based on DeepLabCut
Source: Front Behav Neurosci. 2026 Apr 21;20:1819151. doi: 10.3389/fnbeh.2026.1819151 (PMC13139089; doi:10.3389/fnbeh.2026.1819151)
Supplement: Supplementary file 1 [file Table_1.DOCX]

Supplementary Material

**Supplementary Table**

**Supplementary Table S1 Traditional behavioral indicators**

| Indicator | Definition |
| --- | --- |
| Frequency of exploring a new object by the nose tip (2 cm away from the object) | The frequency of the nose tip entering the area within 2 cm of the bottom edge of the new object's surface |
| Frequency of exploring an old object by the nose tip (2 cm away from the object) | The frequency of the nose tip entering the area within 2 cm of the bottom edge of the old object's surface |
| Duration of exploring a new object by the nose tip (2 cm away from the object) (s) | The duration of the nose tip entering the area within 2 cm of the bottom edge of the new object's surface |
| Duration of exploring an old object by the nose tip (2 cm away from the object) (s) | The duration of the nose tip entering the area within 2 cm of the bottom edge of the old object's surface |
| Frequency preference for exploring objects with the tip of the nose (2 cm away from the object) | The proportion of frequency the nose tip is used as a reference point to enter the area 2 cm outside the bottom edge of the new object's surface, out of the total frequency the nose tip is used as a reference point to enter the area 2 cm outside the bottom edge of both the new and old objects' surfaces. |
| Duration preference for exploring objects with the tip of the nose (2 cm away from the object) | The proportion of duration the nose tip is used as a reference point to enter the area 2 cm outside the bottom edge of the new object's surface, out of the total duration the nose tip is used as a reference point to enter the area 2 cm outside the bottom edge of both the new and old objects' surfaces. |

Notes: This table lists the definitions of traditional and commonly used behavioral indicators in object recognition experiments, which are widely applied in conventional visual observation assays.
