# Supplementary material for: Establishment and preliminary application of object recognition system based on DeepLabCut
Source: Front Behav Neurosci. 2026 Apr 21;20:1819151. doi: 10.3389/fnbeh.2026.1819151 (PMC13139089; doi:10.3389/fnbeh.2026.1819151)
Supplement: Supplementary file 2 [file Table_2.DOCX]

Supplementary Material

**Supplementary Table**

**Supplementary Table S2 Novel behavioral indicators of this system**

| Indicator | Definition |
| --- | --- |
| Frequency of exploring a new object by nose tip (1 cm away from the object) | The frequency of the nose tip entering the area within 1 cm of the bottom edge of the new object's surface |
| Frequency of exploring an old object by nose tip (1 cm away from the object) | The frequency of the nose tip entering the area within 1 cm of the bottom edge of the old object's surface |
| Duration of exploring a new object by nose tip (1 cm away from the object) (s) | The duration of the nose tip entering the area within 1 cm of the bottom edge of the new object's surface |
| Duration of exploring an old object by nose tip (1 cm away from the object) (s) | The duration of the nose tip entering the area within 1 cm of the bottom edge of the old object's surface |
| Frequency of exploring a new object by nose tip(1.5 cm away from the object) | The frequency of the nose tip entering the area within 1.5 cm of the bottom edge of the new object's surface |
| Frequency of exploring an old object by nose tip (1.5 cm away from the object) | The frequency of the nose tip entering the area within 1.5 cm of the bottom edge of the old object's surface |
| Duration of exploring a new object by the nose tip (1.5 cm away from the object) (s) | The duration of the nose tip entering the area within 1.5 cm of the bottom edge of the new object's surface |
| Duration of exploring an old object by nose tip (1.5 cm away from the object) (s) | The duration of the nose tip entering the area within 1.5 cm of the bottom edge of the old object's surface |
| Frequency of exploring a new object at body center (2 cm away from the object) | The frequency of body center entering the area within 2 cm of the bottom edge of the new object's surface |
| Frequency of exploring an old object at body center (2 cm away from the object) | The frequency of body center entering the area within 2 cm of the bottom edge of the old object's surface |
| Duration of exploring a new object at body center (2 cm away from the object) (s) | The duration of body center entering the area within 2 cm of the bottom edge of the new object's surface |
| Duration of exploring an old object at the body center (2 cm away from the object) (s) | The duration of body center entering the area within 2 cm of the bottom edge of the old object's surface |
| Frequency of exploring a new object by nose tip and body center (2 cm away from the object) | The frequency of both the nose tip and body center entering the area within 2 cm of the bottom edge of the new object's surface |
| Frequency of exploring an old object by the nose tip and body center(2 cm away from the object) | The frequency of both the nose tip and body center entering the area within 2 cm of the bottom edge of the old object's surface |
| Duration of exploring a new object by nose tip and body center (2 cm away from the object) (s) | The duration of both the nose tip and body center entering the area within 2 cm of the bottom edge of the new object's surface |
| Duration of exploring an old object by nose tip and body center (2 cm away from the object) (s) | The duration of both the nose tip and body center entering the area within 2 cm of the bottom edge of the old object's surface |
| Frequency of touching a new object by nose tip | The frequency of the nose tip touching the bottom edge of the new object's surface |
| Frequency of touching an old object by nose tip | The frequency of the nose tip touching the bottom edge of the old object's surface |
| Duration of touching a new object by nose tip (s) | The duration of the nose tip touching the bottom edge of the new object's surface |
| Duration of touching an old object by nose tip (s) | The duration of the nose tip touching the bottom edge of the old object's surface |
| Frequency preference for exploring objects with the tip of the nose (1 cm away from the object) | The proportion of frequency the nose tip is used as a reference point to enter the area 1 cm outside the bottom edge of the new object's surface, out of the total frequency the nose tip is used as a reference point to enter the area 1 cm outside the bottom edge of both the new and old objects' surfaces. |
| Duration preference for exploring objects with the tip of the nose (1 cm away from the object) | The proportion of duration the nose tip is used as a reference point to enter the area 1 cm outside the bottom edge of the new object's surface, out of the total duration the nose tip is used as a reference point to enter the area 1 cm outside the bottom edge of both the new and old objects' surfaces. |
| Frequency preference for exploring objects with the tip of the nose (1.5 cm away from the object) | The proportion of frequency the nose tip is used as a reference point to enter the area 1.5 cm outside the bottom edge of the new object's surface, out of the total frequency the nose tip is used as a reference point to enter the area 1.5 cm outside the bottom edge of both the new and old objects' surfaces. |
| Duration preference for exploring objects with the tip of the nose (1.5 cm away from the object) | The proportion of duration the nose tip is used as a reference point to enter the area 1.5 cm outside the bottom edge of the new object's surface, out of the total number of duration the nose tip is used as a reference point to enter the area 1.5 cm outside the bottom edge of both the new and old objects' surfaces. |
| Frequency preference for exploring objects with the tip of the body center (2 cm away from the object) | The proportion of frequency the body center is used as a reference point to enter the area2 cm outside the bottom edge of the new object's surface, out of the total frequency the body center is used as a reference point to enter the area 2 cm outside the bottom edge of both the new and old objects' surfaces |
| Duration preference for exploring objects with the tip of the body center (2 cm away from the object) | The proportion of duration the body center is used as a reference point to enter the area 2 cm outside the bottom edge of the new object's surface, out of the total number of duration the body center is used as a reference point to enter the area 2 cm outside the bottom edge of both the new and old objects' surfaces. |
| Frequency preference for exploring objects with the tip of the nose and body center (2 cm away from the object) | The proportion of frequency the nose tip and body center is used as a reference point to enter the area 2 cm outside the bottom edge of the new object's surface, out of the total frequency the nose tip and body center is used as a reference point to enter the area 2 cm outside the bottom edge of both the new and old objects' surfaces |
| Duration preference for exploring objects with the tip of the nose and body center (2 cm away from the object) | The proportion of duration the nose tip and body center is used as a reference point to enter the area 2 cm outside the bottom edge of the new object's surface, out of the total number of duration the nose tip and body center is used as a reference point to enter the area 2 cm outside the bottom edge of both the new and old objects' surfaces |
| Frequency preference for the tip of the nose touching object | The proportion of frequency the nose tip touches the bottom edge of the new object's surface, out of the total frequency the nose tip touches the bottom edges of both the new and old objects' surfaces. |
| Duration preference for the tip of the nose touching object | The proportion of duration the nose tip touches the bottom edge of the new object's surface, out of the total duration the nose tip touches the bottom edges of both the new and old objects' surfaces |

Notes: This table presents the definitions of novel refined behavioral indicators constructed based on DeepLabCut. The indicators are classified by calibration points (nose tip, body center, nose tip + body center) and distance thresholds (1 cm, 1.5 cm, 2 cm, direct contact), covering a total of 30 indicators.
