# Supplementary material for: Establishment and preliminary application of object recognition system based on DeepLabCut
Source: Front Behav Neurosci. 2026 Apr 21;20:1819151. doi: 10.3389/fnbeh.2026.1819151 (PMC13139089; doi:10.3389/fnbeh.2026.1819151)
Supplement: Supplementary file 3 [file Table_3.DOCX]

Supplementary Material

**Supplementary Table**

**Supplementary Table S3 The results of comparison between manual visual observation and NOR analysis system**

| Indicator | Visual observation | NOR analysis system | *p* |
| --- | --- | --- | --- |
| Frequency of exploring a new object by the nose tip (2 cm away from the object) | 19.43±5.23 | 21.71±5.99 | ＞0.05 |
| Frequency of exploring an old object by the nose tip (2 cm away from the object) | 15.14±4.76 | 16.26±4.59 | ＞0.1 |
| Duration of exploring a new object by the nose tip (2 cm away from the object) (s) | 23.54±5.01 | 26.23±8.43 | ＞0.1 |
| Duration of exploring an old object by the nose tip (2 cm away from the object) (s) | 16.28±6.09 | 17.34±6.60 | ＞0.1 |
| Frequency preference for exploring objects with the tip of the nose (2 cm away from the object) | 0.56±0.05 | 0.57±0.08 | ＞0.05 |
| Duration preference for exploring objects with the tip of the nose (2 cm away from the object) | 0.60±0.06 | 0.61±0.04 | ＞0.1 |

Notes: This table shows the comparative data of detection results for traditional nose tip exploration indicators (2 cm threshold) between manual visual observation and the NOR analysis system. Data are presented as mean±SD.The data conformed to a normal distribution; therefore,the paired-sample t-test was employed for analysis；*p*＞0.05and *p*＞0.1 vs visual observation.
